# Detection and identification of single ribonucleotide monophosphates using a dual in-plane nanopore sensor made in a thermoplastic *via* replication†

**DOI:** 10.1039/d3lc01062g

**Published:** 2024-05-14

**Authors:** Chathurika Rathnayaka, Indu A. Chandrosoma, Junseo Choi, Katie Childers, Maximillian Chibuike, Khurshed Akabirov, Farhad Shiri, Adam R. Hall, Maxwell Lee, Collin McKinney, Matthew Verber, Sunggook Park, Steven A. Soper

**Affiliations:** a Department of Chemistry, The University of Kansas, Lawrence, KS 66045, USA; b Center of BioModular Multiscale Systems for Precision Medicine, USA; c Mechanical & Industrial Engineering Department, Louisiana State University, Baton Rouge, LA 70803, USA; d Virginia Tech-Wake Forest School of Biomedical Engineering and Sciences, Wake Forest School of Medicine, Winston Salem, NC 27101, USA; e Atrium Wake Forest Baptist Comprehensive Cancer Center, Wake Forest School of Medicine, Winston Salem, NC 27157, USA; f Department of Chemistry, University of North Carolina, Chapel Hill, Chapel Hill, NC 27599, USA; g Department of Mechanical Engineering, The University of Kansas, Lawrence, KS 66045, USA; h Bioengineering Program, The University of Kansas, Lawrence, KS 66045, USA; i KU Cancer Center, University of Kansas Medical Center, Kansas City, KS 66160, USA

## Abstract

We report the generation of ~8 nm dual in-plane pores fabricated in a thermoplastic *via* nanoimprint lithography (NIL). These pores were connected in series with nanochannels, one of which served as a flight tube to allow the identification of single molecules based on their molecular-dependent apparent mobilities (*i.e.*, dual in-plane nanopore sensor). Two different thermoplastics were investigated including poly(methyl methacrylate), PMMA, and cyclic olefin polymer, COP, as the substrate for the sensor both of which were sealed using a low glass transition cover plate (cyclic olefin co-polymer, COC) that could be thermally fusion bonded to the PMMA or COP substrate at a temperature minimizing nanostructure deformation. Unique to these dual in-plane nanopore sensors was two pores flanking each side of the nanometer flight tube (50 × 50 nm, width × depth) that was 10 μm in length. The utility of this dual in-plane nanopore sensor was evaluated to not only detect, but also identify single ribonucleotide monophosphates (rNMPs) by using the travel time (time-of-flight, ToF), the resistive pulse event amplitude, and the dwell time. In spite of the relatively large size of these in-plane pores (~8 nm effective diameter), we could detect *via* resistive pulse sensing (RPS) single rNMP molecules at a mass load of 3.9 fg, which was ascribed to the unique structural features of the nanofluidic network and the use of a thermoplastic with low relative dielectric constants, which resulted in a low RMS noise level in the open pore current. Our data indicated that the identification accuracy of individual rNMPs was high, which was ascribed to an improved chromatographic contribution to the nano-electrophoresis apparent mobility. With the ToF data only, the identification accuracy was 98.3%. However, when incorporating the resistive pulse sensing event amplitude and dwell time in conjunction with the ToF and analyzed *via* principal component analysis (PCA), the identification accuracy reached 100%. These findings pave the way for the realization of a novel chip-based single-molecule RNA sequencing technology.

## Introduction

Advancements in transcriptomics are creating opportunities in translational and basic research through the elucidation of RNA structure, which has mainly been spawned by next generation sequencing (NGS),^1,2^ in spite of the fact that several technical challenges associated with NGS remain.^3,4^ Most of these challenges are associated with library preparation, which requires the RNA to be converted into cDNA using reverse transcription followed by amplification that aids in detection.^3^ However, the amplification step can introduce biases and other artifacts such as the loss of important RNA epitranscriptomic information.^5,6^

Single-molecule sequencing using nanopore-related technologies can address many of the aforementioned challenges.^7,8^ For example, single-molecule nanopore sequencing in some cases can eliminate the need for amplification and provide longer reads compared to NGS; the elimination of amplification and reverse transcription can preserve the integrity of post-transcriptionally modified ribonucleotides. While nanopore sequencing has seen some attractive improvements in its figures-of-merit, these platforms still require inputs (~1 μg) that can necessitate the need for amplification.

The transduction modality used in nanopore sequencing is resistive pulse sensing (RPS) and consists of moving a single molecule through a biological nanopore with the sequence read by monitoring changes in the ionic current as nucleotides pass through the pore.^9^ These pores are typically embedded within a thin membrane separating two electrolyte chambers (*i.e.*, out-of-plane nanopores).^10^ In the measurement approach, carrier electrolyte ions are driven through an electrically biased nanopore while simultaneously monitoring the current (*i.e.*, open pore current, $I_{0}$). When a single molecule is present in the nanopore, it will disrupt $I_{0}$ resulting in a measurable change in the current (ΔI). Generally, $I_{0}$ and ΔI at relatively high ionic strengths (≥0.1M) can be determined using;^11^

(1) $$I_{0}=V\left(\left[\mu_{+}+\mu_{-}\right]n_{\text{salt}}e+\mu_{+}4\sigma /d_{\text{pore}}\right){\left(4h_{\text{eff}}/\pi d^{2}+1/d\right)}^{-1}$$

(2) $$\text{Δ}I=I_{0}-I_{\text{target}}\;=V\left(\left[\mu_{+}+\mu_{-}\right]n_{\text{salt}}e+\mu_{+}4\sigma /d_{\text{pore}}\right){\left(4h_{\text{eff}}/\pi d_{\text{eff}}^{2}+{1/d}_{\text{eff}}\right)}^{-1}$$

where *σ* is the surface charge density, *V* is the applied voltage, $\mu_{\pm }$ is the mobility of cations and anions, $n_{\text{salt}}$ is the number density of salt ions, *e* is the fundamental electron charge, $h_{\text{eff}}$ is the length (or thickness) of the nanopore, *d* is the diameter of the pore assuming that the nanopore is cylindrically shaped, and *d*_eff_ is the effective diameter of the pore. According to eqn (2), Δ*I* can be increased by using an electrolyte solution with high ionic strength^12–14^ and/or by reducing the diameter^15,16^ or length^11,17^ of the nanopore.

Solid-state nanopores were developed to address challenges associated with the use of biological nanopores.^18–25^ Compared to biological nanopores, solid-state nanopores have tunable pore sizes, possess the ability to modify the surface chemistry of the pore, and have greater potential for integration into lab-on-a-chip systems.^9,26,27^ Focused ion beam (FIB),^28–30^ electron microscopy (EM), and dielectric breakdown^31,32^ are typically employed to fabricate solid-state nanopores.^20^ However, no report has appeared in which solid-state nanopores have been used for *de novo* sequencing of either RNA or DNA.

Nanopores, which can in principle be used to detect small molecules such as nucleotides and amino acids, can be categorized according to the pore orientation relative to the substrate; out-of-plane or in-plane.^33–35^ Solid state nanopores fabricated in planar substrates (molecular translocation parallel to the substrate surface) are known as “in-plane” nanopores.^36^ Advantages of in-plane nanopores include improved mass transfer of analytes to the nanopore,^33^ and new measurement modalities. For example, Harms *et al.*^37^ fabricated a silicon nanochannel device *via* direct ion beam milling with two in-plane nanopores (width = 50 nm, depth = 50 nm and length = 40 nm) placed in series (2 μm spacing) separated by a drift channel to determine the electrophoretic mobility of translocating species.

In previous work,^38^ we used two in-plane nanopores placed in series separated by a 5 μm nanochannel fabricated in poly(ethylene)glycol diacrylate (PEGDA) using UV-nanoimprint lithography (UV-NIL) for the identification of deoxynucleotide monophosphates (dNMPs) based on their characteristic molecular dependent time-of-flight (ToF), which is the time a single molecule will take to travel from one pore to the other nanopore and is dependent on the apparent electrophoretic mobility of a molecule. The authors showed identification accuracies of dNMPs using their molecular-dependent ToF of ~94%.^39^ However, PEGDA is a hydrogel making it difficult to maintain a consistent nanopore size over time in aqueous media due to swelling effects. In addition, only the ToF was used for identification of the dNMPs and no other RPS parameters, such as the event amplitude or the event dwell time, which represents the translocation time of an analyte through the nanopore, were used. In addition, the use of PEGDA does not lend itself to high scale production modalities, such as injection molding, to make the platform conducive to commercial applications.

Polydimethylsiloxane (PDMS) in-plane nanopores have also been reported for RPS.^40,41^ For example, Sohn and team developed a PDMS-based in-plane sensor to measure single DNA molecules.^42^ In this example, a SU-8 relief was made using a combination of photolithography (microstructures) and electron-beam lithography (nanostructures) from which replicas could be generated using soft lithography. Unfortunately, PDMS has challenges such as the instability of its surface chemistry following O_2_ plasma activation to make it more hydrophilic, and its inability to be injection molded meaning that the device production rate is low and does not scale well with number of devices in terms of cost.^43^

A class of materials that can address the aforementioned challenges are thermoplastics, which are branched polymers capable of softening when heated and hardening when cooled, and can be used to fabricate in-plane nanopore sensors *via* replication technologies.^15,39,44,45^ The advantages of thermoplastics include the ability to tailor the in-plane nanopore size by changing the thermal fusion bonding pressure.^15^ Also, thermoplastic surface chemistry can be easily controlled using techniques such as O_2_ plasma or UV/O_3_ (ref. 46) to generate –COOH groups to make the surface more wettable.^15,47–49^ Finally, RPS devices using thermoplastics require photolithography and focused ion beam milling to produce a silicon master mold from which a number of replicas can be generated, significantly reducing device cost and increasing production rate.^50–52^ For example, thermoplastic RPS devices can be fabricated using nano-injection molding, which has the ability to produce devices at high production rates.^43,53,54^

**Fig. 1:**
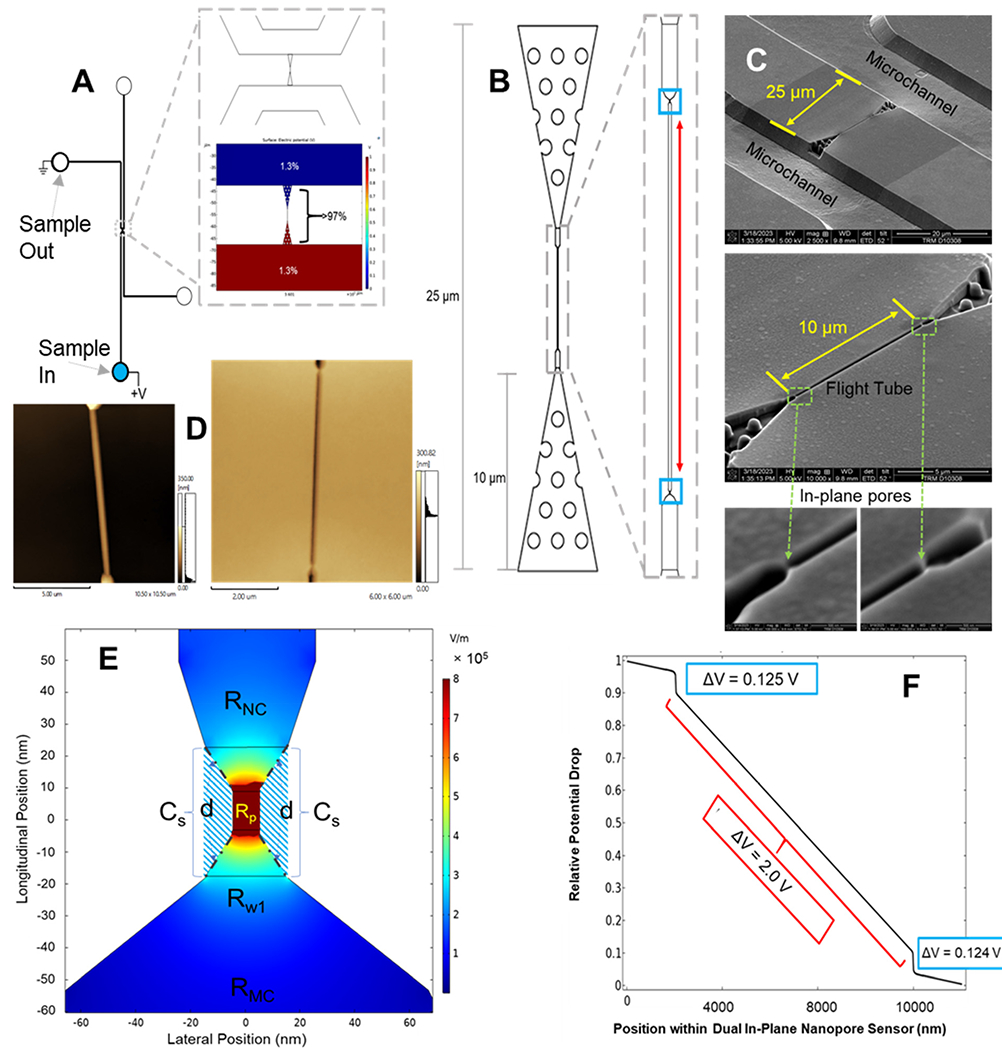
(A) Schematic showing the layout of the mixed-scale fluidic circuit including the microchannels and dual in-plane nanopore sensor. Also shown is a COMSOL simulation indicating the relative voltage drop throughout the fluidic circuit. There are four reservoirs (two on either side of the dual in-plane nanopore sensor) showing the sample inlet and outlet reservoirs. (B) 2D schematic of the dual in-plane nanopore sensor, which consisted of a 3D tapered input populated with pillars used to help electrokinetically shuttle single molecules into the sensor from the microchannels. There were in-plane nanopores flanking either side of the flight tube used to determine the molecular-dependent apparent electrophoretic mobility of the particular molecule translocating through the sensor. The pores had a pseudo-Gaussian shape determined by the ion beam intensity profile and the flight tube had a square shape. (C) SEM images of the Si master mold of the dual in-plane nanopore sensor (upper panel). In this SEM, the microchannels are shown as well. A high-resolution SEM of the dual in-plane nanopore sensor is shown in the middle panel with the 10 μm length flight tube. The lower panels show high resolution SEMs of the two in-plane pores that flank the flight tube. These pores are both 10 nm in length. (D) AFM images of the dual in-plane nanopores that flanked the nanometer flight tube. The AFM images are those for the resin stamp (left) and the imprinted device (right). (E) COMSOL simulation of the relative voltage drop through an in-plane pore. (F) Relative voltage drop through the sensor as a function of sensor position. The absolute voltage drop through each element of the sensor could be determined by multiplying the relative potential drop × applied voltage to the sensor. The flight tube had a length of 10 μm. *C*_s_ = capacitance of the polymer substrate (cross-hashed area; dot-dashed line shows capacitor plates, *d* = effective distance between plates), *R*_NC_ = nanochannel flight tube resistance, and *R*_MC_ = microchannel resistance.

In this work, we report a dual in-plane nanopore sensor (see Fig. 1A) made in a thermoplastic (PMMA or COP) *via* replication for the detection and identification of ribonucleotide monophosphates (rNMPs). We recently reported the surface immobilization of a processive exoribonuclease (XRN1) to a thermoplastic that produce rNMPs from single RNA molecules that serves as a foundational piece to build an innovative RNA sequencing technology.^55^ The work reported herein provides another step toward realizing this technology. Our sensor consists of a 3D tapered input with pillars to extend the electric field into the adjoining microchannel to improve capture,^56^ and 2 in-plane nanopores positioned in series that flank both ends of a flight tube from which the ToF can be deduced to aid in single-molecule identification (Fig. 1B). From this device, we could determine the apparent electrophoretic mobility, which we will show is unique for each rNMP. We used the dual in-plane nanopore sensor with a 10 μm flight tube length to identify rNMPs based on multiple RPS variables including the dwell time, current transient amplitude, and ToF in conjunction with principle component analysis (PCA) to improve identification accuracy of single rNMP molecules.

In Fig. 1C is shown SEMs of the Si master mold used to generate resin stamps *via* UV-NIL. The plastic device was then made from the resin stamp *via* thermal NIL – AFM pictures of both the resin stamp and imprinted device are shown in Fig. 1D.

As seen from Fig. 1A, 3% of the fluidic circuit voltage drop occurred in the microchannels while 97% of the voltage drop occurred in the dual in-plane nanosensor region of this chip based on the physical dimensions of this chip (see Table S1†). Fig. 1E provides a 2D COMSOL simulation (see Table S1† for COMSOL variables) of the relative voltage drop around the nanopore. Inspection of Fig. 1F shows a relatively high electric field strength in the nanochannel between the in-plane pores to avoid drifting effects that would produce high variability in the ToF determinations due to drifting effects.

## Results and discussion

### Nanofluidic device fabrication, assembly, and electrical characteristics

Nanofluidic devices were fabricated in a thermoplastic using strategies we have reported,^51,57^ which briefly consists of making microstructures *via* photolithography and nanostructures by focused ion beam milling in Si followed by producing resin stamps *via* UV-NIL and production of the final device *via* thermal NIL (see Fig. S1 and the ESI† for more fabrication details). The in-plane nanopores were positioned at either end of a nanochannel, which was 10 μm in length and 50 × 50 nm in width and depth (see Fig. 1C and D). SEM inspection of the resin stamp indicated the average height of the in-plane nanopores were 24.3 ± 2.0 nm (*n* = 4) and the depth of the nanopores in the imprinted substrate were 25.4 ± 1.7 nm (*n* = 3) indicating good replication fidelity.^58^ For this work, we used TPGDA (tripropyleneglycol diacrylate) resin stamps because we could produce >20 replicas from a single stamp *via* thermal NIL without noticing any damage imposed on the resin stamp. Recent work by our team indicated that TPGDA yielded the best results in terms of replication fidelity *via* UV-NIL from a Si master mold.^58^ Following noticeable damage to the resin stamp, we could make another resin stamp using UV-NIL from the Si master mold. In addition, thermal NIL from the resin stamp permitted the use of different thermoplastics.

The in-plane nanopore signal bandwidth can be calculated using a similar formalism as presented by Uram *et al.* for out-of-plane nanopores, where the bandwidth (*f*_c_; Hz) can be calculated from;^59^

(3) $$f_{\text{C}}<\frac{1}{2\pi R_{\text{T}}C_{\text{T}}}$$

where *R*_T_ is equal to *R*_w1_ + *R*_NC_ + *R*_w2_ (see Fig. 1E and S2A†) and *C* is the capacitance of the polymer extending from the substrate to form the in-plane pore (see Fig. 1C and E). In Fig. S2B† is the equivalent electric circuit for the sensor shown in Fig. 1B. The total resistance (*R*_T_) was calculated from the relative voltage drop in each region of the device, the applied voltage (2.5 V) and the total current in the circuit (8.4 nA) and was found to be 313 MΩ (see Fig. S2C†). We estimated *C*_T_ (Fig. 1E) by determining the capacitance from each pore (*C*_S_), the relative dielectric constant of the plastic material (COP, *ε*_r_ = 2.2; PMMA, *ε*_r_ = 3.9), the area (*A*) of the capacitor plates (4000 nm^2^) and the average distance between the plates (*d*, 10 nm; see ESI†; for calculations). The pore bandwidth was estimated to be 130 MHz for COP and 73 MHz for PMMA; the difference in the pore bandwidth resulted from differences in the relative dielectric constant for both materials. The AxoPatch current amplifier used in these experiments was operated at 10 kHz (low pass filter; sets the bandwidth) indicating that the pores do not limit the operational bandwidth. As a matter of comparison, the bandwidth of an out-of-plane pore suspended in a silicon nitride membrane was 32 MHz while a PET pore was 16 MHz.^59^ The higher bandwidth for our in-plane pores is due primarily to the smaller area of the capacitor plates used herein.

### Damage of thermoplastic nanostructures using O_2_ plasma or UV/O_3_ irradiation

In the process of assembling thermoplastic nanofluidic devices, the substrate and cover plate required exposure to either UV/O_3_ or O_2_ plasma before thermal fusion bonding to optimize the process yield rate.^60,61^ This step also increases the wettability of the plastic (*i.e.*, increase surface energy), which is critical for RPS readout because it can reduce nanobubble formation within the nanopores that can contribute to 1/*f* noise.^59^ For microstructures, the effects of O_2_ plasma or UV/O_3_ irradiation are inconsequential, but can be an issue for nanofluidic devices due to surface damage that can affect the integrity of the replicated nanostructures. As we have shown, PMMA and COP or COC can be activated with UV/O_3_ exposure and after ~15 min (22 mW cm^−2^), the polymers show stable water contact angles (37° and 32° for PMMA and COC or COP, respectively).^60,62^ COC and COP show similar trends when exposed to O_2_ plasma or UV/O_3_ in terms of reduction in their water contact angles.^63^

**Fig. 2:**
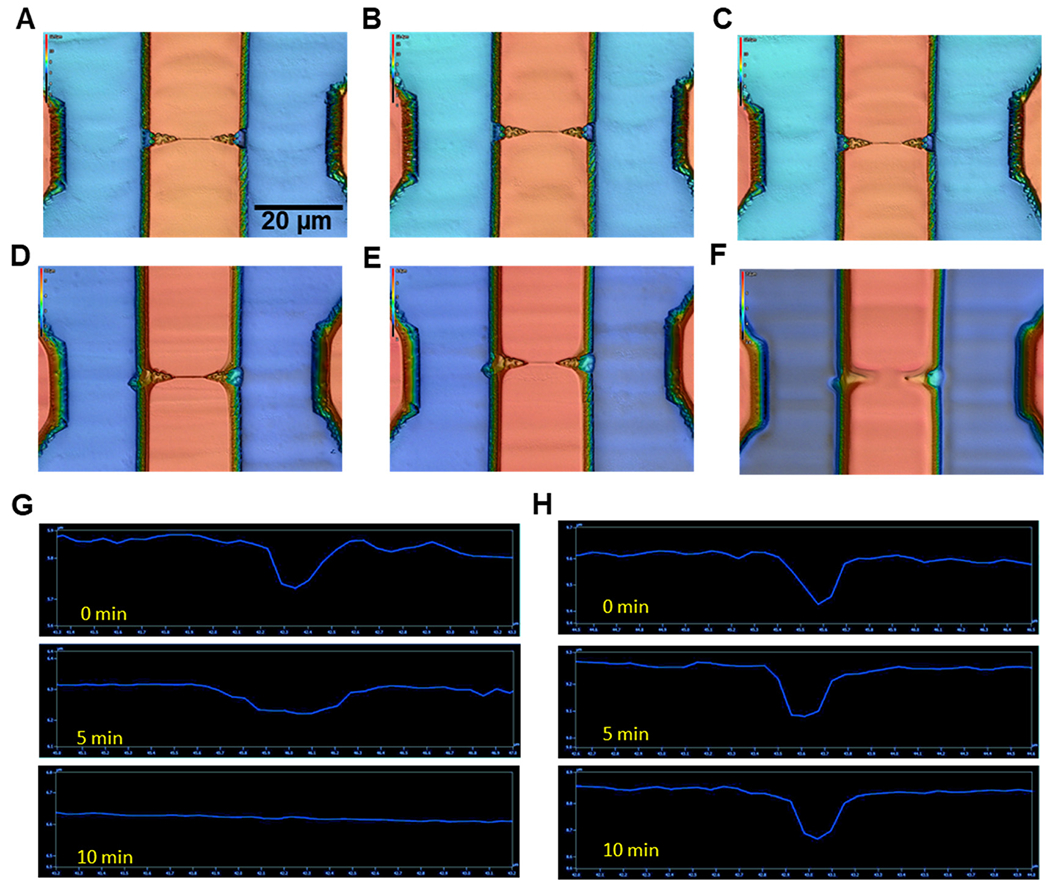
Rapid scanning confocal images of dual in-plane nanopore thermoplastic devices (150× objective; *λ* = 405 nm). (A) Unexposed, (B) 5 min O_2_ plasma exposed, and (C) 10 min UV/O_3_ exposed COP devices. (D) Unexposed, (E) 5 min O_2_ plasma activated, and (F) 10 min UV/O_3_ exposed PMMA nanofluidic devices. Depth profile of (G) PMMA and (H) COP nanochannels (50 × 50 nm; width × depth) for different UV/O_3_ exposure times. UV/O_3_ intensity = 20 mW cm^−2^. For (B–F), the scale bar shown in (A) is applicable.

The effects of UV/O_3_ exposure times (0, 5, 10 min) on the integrity of replicated nanostructures was explored using nanofluidic devices imprinted in PMMA or COP substrates. There were clear signs of nanostructure damage of PMMA, but not COP (see Fig. 2A–F). In the case of O_2_ plasma exposure, both PMMA and COP nanofluidic structures did not show any signs of damage irrespective of exposure time (data not shown). PMMA nanofluidic structures exhibited damage after 5 min of UV/O_3_ exposure (Fig. 2G and H). COP nanofluidic structures did not show any signs of damage even after 10 min of UV/O_3_ exposure. It has been shown that for PMMA, photo-fragmentation does occur under UV exposure.^64^ In that study, the authors showed scission of both side and main chains after photolysis with a UV source. PMMA can absorb UV light that can induce photo-fragmentation. However, COP is a saturated alkane and would be expected to have very low if any absorption in the near UV that would result in photo-fragmentation. Therefore, in the remaining sections of this study, we used COP as the nanofluidic substrate with UV/O_3_ exposure and PMMA with O_2_ plasma exposure to affect the proper surface chemistry without damaging the nanostructures.

### Optimal design of dual in-plane nanopore sensor

**Fig. 3:**
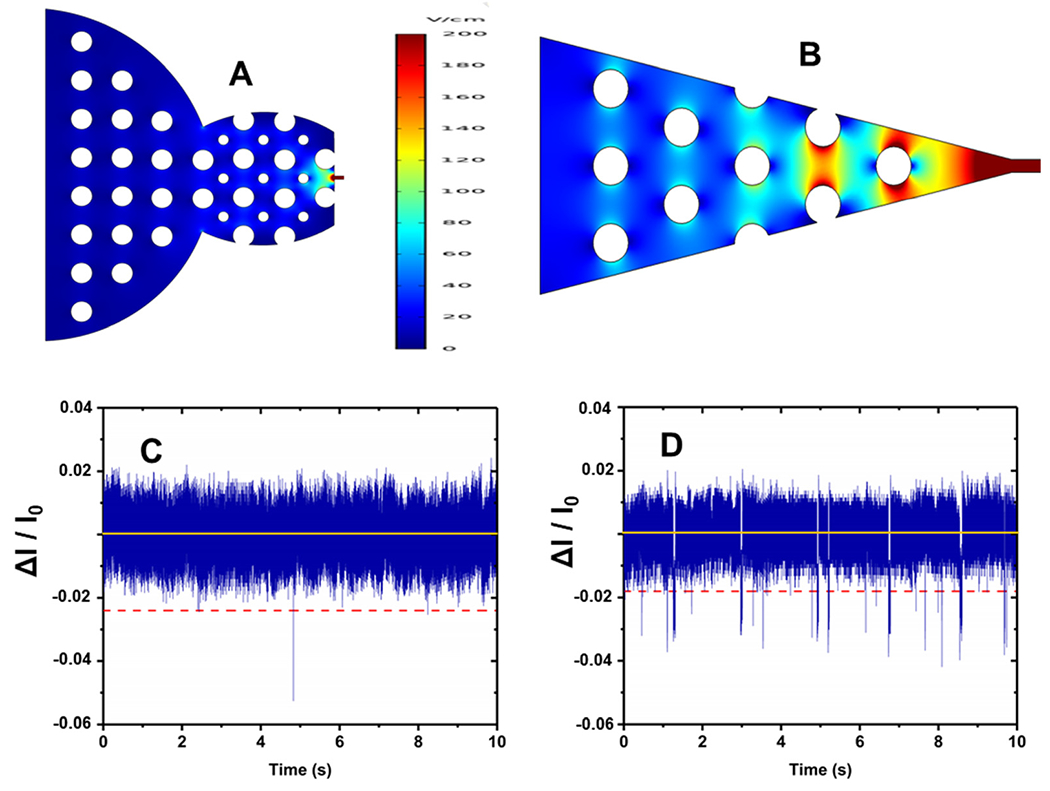
Two different dual in-plane nanopore sensor designs. COMSOL simulations showing the electric field strength distribution for; (A) blunt end input geometry and (B) taper input geometry. Label-free detection of rCMPs (10 nM) using a PMMA/COC dual in-plane nanopore sensor. A 10 s transient current trace obtained with: (C) blunt end geometry; and (D) tapered end geometry. Δ*I*/*I*_0_ represents the ratio of the RPS event amplitude normalized with respect to the open pore current (negative values are indicative of a negative polarity event – current value decreases for an RPS event).

RPS of rNMPs was tested using two different in-plane nanopore sensor designs as shown in Fig. 3A and B. One geometry had a blunt end interface between the microchannel and the dual in-plane nanopore sensor, while the second design had a 3D tapered interface. We performed translocation experiments with rNMPs to determine the capture rate of single rNMPs into each sensor design. The observed event frequency for rNMPs with a blunt end geometry (Fig. 3C) was 1 event per 10 s, which was lower compared to the tapered geometry (Fig. 3D) that produced 24 events per 10 s. For the blunt end geometry, the width and depth of the microchannels near the blunt inlet were 130 μm and 10 μm, respectively. However, in the tapered iteration, we reduced the width and depth of the microchannels near the funnel inlet to 25 μm and 3 μm, respectively. This allowed for better capture rates due to better extension of the electric field into the adjoining microchannel offered by the tapered input (see Fig. 3A and B). At an rNMP concentration of 10 nM and a loading channel volume of 1.12 nL, the mass of rNMPs loaded into the sensor was 11.2 amols (3.9 fg). Therefore, placement of a 3D taper geometry and a smaller adjoining microchannel can improve stochastic capture rates for in-plane resistive pulse sensors.

### ToF measurements of single molecules: concentration considerations

As seen in Fig. 1A, in-plane nanopores flanked a nanometer channel (50 × 50 nm; width × depth; length = 10 μm; see Fig. S3A† for a high-resolution SEM of the in-plane pore and was ~25 nm), which served as a flight tube to determine the apparent electrophoretic mobility of a single molecule. The apparent mobility was calculated from the time difference (*i.e.*, ToF) between a pair of current transient peaks with a known distance between the pores and normalized with respect to the electric field strength in the flight tube.

We have found that the use of low concentrations of targets reduces the relative standard deviation (RSD) in ToF determinations because of a lower probability of multiple occupancy of molecules within the flight tube.^15^ Therefore, it was important to make sure a peak pair resulted from a single molecule and not to a second molecule entering the flight tube before the first one successfully exited the flight tube. For these experiments, we used an rNMP concentration of 100 or 10 nM with a carrier electrolyte consisting of 1× NEBuffer 3 (pH = 7.9), which is a buffer compatible with XRN1 enzyme activity. XRN1 is an exoribonuclease used to processively clip intact RNA molecules in the 5′ → 3′ direction generating rNMPs.^55^ At a concentration of 100 nM, the single molecule occupancy within the 10 μm nanometer flight tube (volume = 18.3 aL) was 1.1. The single-molecule occupancy (*P*_SM_) was calculated from;^54^

$$P_{\text{SM}}=N_{\text{A}}P_{\text{V}}\left[\text{C}\right]$$

where *N*_A_ is Avogadro’s number, *P*_V_ is the probe volume of the sensor, which includes the two in-plane pores and the flight tube, and [C] is the concentration of the analyte. For a Poisson distribution of single molecule occupancies within the flight tube, 36.7% of the peak pairs are due to single molecule events while 20.2% peak pairs could arise from double occupancy. At a concentration of 10 nM, the percentage of peak pairs due to single molecule events in the 10 μm flight tube is 9.9% and 0.54% for double occupancy events. As seen from these simple calculations, there is a 1.8-fold difference between the percentage of single and double occupancy events for the 100 nM rNMP solution, but there is an 18.3-fold difference for the 10 nM concentration indicating higher confidence that the ToF measured is due to a single molecule. As a final note, the single-molecule occupancy for a single in-plane nanopore (volume ~1 zL) was calculated to be 1.4 × 10^−6^ (concentration = 10 nM) and thus, the occupancy of two molecules resident within the in-plane pore sensing volume at any given time is infinitesimally small.

### RPS of single rNMPs using the dual in-plane nanopore sensor

In our recent publication, we reported the nanoscale electrophoresis of fluorescently labeled rNMPs using 100 × 100 nm (depth × width) thermoplastic nanochannels.^44^ In this study, we used a label-free approach for the detection of rNMPs using PMMA/COC or COP/COC dual in-plane nanopore sensors and thermally fusion bonded the substrate to the cover plate at 170 psi for 15 min. The nanopores are hemi-circular due to the Gaussian intensity profile of the ion beam used to fabricate the pores in the Si master mold that was transferred to the thermoplastic *via* thermal NIL and a flat cover plate used to enclose the fluidic network.^15^ As noted above, two in-plane pores were placed in series with a fixed distance between the pores and a known applied voltage, which allowed for elucidation of a single molecule’s apparent electrophoretic mobility. The apparent mobility represents the vector sum of the electroosmotic flow of the device and the characteristic electrophoretic mobility of the rNMP.^37,38^ Inspection of Fig. 3D indicated an RPS event with an average dwell time of 340 μs and amplitude of 141 pA and a ToF of 5.5 ms for rCMP.

The ToFs of the rNMPs were deduced from paired peaks within the RPS data trace that were identified using three selection criteria:^38^ (i) the amplitudes of both peaks comprising a pair should be >3× the RMS noise of *I*_0_. *I*_0_ of the dual in-plane nanopore device was 8.4 ± 1.9 nA (inter-device; Fig. S3B†). The RMS noise of the open pore current of the 170 psi bonded device using a 1× NEBuffer (*V* = 2.5 V) was found to be 19.5 pA and therefore, only peaks with amplitudes >58.5 pA was scored as events. (ii) The minimum ToF for a single rNMP molecule should be greater than the dwell time (peak width) of each current transient peak of the pair. (iii) The maximum ToF should be within 1.5 times the ToF calculated from the corrected mobilities of fluorescently labeled rNMPs (removal of mobility of the dye) in nanoscale electrophoresis reported by our group.^44^ As was seen in Choi *et al.*^38^ and Athapattu *et al.*,^15^ unpaired peaks were observed in single-molecule translocation experiments. Unpaired peaks could arise from the use of shallow U-shaped in-plane nanopores, which may result in variations in the RPS amplitude due to molecular translocation occurring at different locations within the nanopore and thus some events do not exceed the threshold condition.^38^ Fast translocation of molecules through nanopores is another possible reason for missing peaks that can generate peak clipping for events exceeding the current amplifier bandwidth. Our current amplifier (Axopatch 200b) in these experiments was operated at 10 kHz. Estimation of the rise time from system bandwidth was calculated using;

(5) Tr=0.35/BW

where Tr is the signal rise time (s), which is defined as the time for the peak amplitude to rise from 10% to 90% of its full amplitude, and BW is the system bandwidth (Hz). Therefore, a BW of 10 kHz allows the system to pass without clipping signals with rise times greater than 35 μs. The experimental signal rise time was determined by inspection of Fig. 3D and found to be 43.4 μs, in close agreement to that predicted from eqn (5) and the 10 kHz low pass filter setting.

### Nanopore scaling effects for the RPS detection of single rNMPs

The size of our sensing nanopore is large relative to previous reports for the RPS detection of single nucleotides using α-hemolysin pores fitted with a β-cyclodextrin moiety to reduce its effective size.^65^ Considering volume displacement effects (*i.e.*, Coulter principle) only would suggest that we should not be able to detect single rNMP with our in-plane pores. However, due to the complexity of our mixed-scale fluidic element, which consists of nanochannels and two in-plane pores placed in series, other physics must be considered to explain the nature of our sensor response. For example, Lee *et al.* demonstrated that Δ*I* for single-molecule RPS events can be significantly enhanced using a guiding nanochannel in series with a single sensing nanopore due to compartmental limitations on ion transport as well as drag forces exerted on the translocating molecule induced by the EOF in the nanochannel.^66^ We also anticipate that some amount of ion selectivity may be at play in our highly charged nanopores and that the positioning of two such elements in series could induce non-linearity in the ion distributions at each sensing pore, providing a localized signal enhancement.^67–69^ Finally, while not expected to be dominant in our system, capacitive effects^70^ may play an additional role in enhancing signals. Extensive modeling of our system using molecular dynamic simulations to better understand these effects is the subject of ongoing studies.

In addition, other reasons could account for our ability to detect single rNMP molecules using our in-plane pores; (i) the extended length of the in-plane pore compared to the α-hemolysin pore resulting in longer dwell times and thus, less event distortion based on bandwidth considerations. (ii) In these experiments, we used a carrier electrolyte of 1× NEBuffer 3, which is comprised of 100 mM NaCl, 50 mM Tris-HCl, 10 mM MgCl_2_, and 1 mM DTT (pH = 7.9). Typically, nanopore measurements are made in the presence of 1 M KCl (*i.e.*, high salt concentration). However, Smeets *et al.* has noted that better RPS signal-to-noise ratios occur when using low salt concentrations with pores containing relatively large sizes.^71^ (iii) The absence of protonation/deprotonation within the in-plane pores, which can contribute to 1/*f* noise in biological pores. We are operating at a pH above the p*K*_a_ of the surface carboxyl groups (p*K*_a_ ~ 5.0).^49^ (iv) The mechanical stability of the in-plane pores is higher compared to pores suspended on thin membranes, helping to reduce 1/*f* noise. (v) The use of a thick and highly insulating dielectric substrate (PMMA or COP), which can reduce thermal voltage noise arising from the pore material’s parasitic capacitance and dielectric loss tangent. Indeed, Lee *et al.* were able to generate sub-pA RMS noise levels in *I*_0_ for PET pores of ~1.5 nm diameter.^17^ For our plastic in-plane pores, the RMS in *I*_0_ was 19.5 and 11 pA for PMMA and COP, respectively.

We also determined the noise characteristics of our devices using a power spectral density (PSD) analysis (see Fig. S4†). We discovered that both PMMA and COP in-plane pore devices generated lower noise characteristics at frequencies below 1000 Hz, where 1/*f* and white noise dominate, due to better mechanical stability of the pore, minimal nanobubble formation, and less surface functional group protonation/deprotonation compared to solid-state silicon nitride nanopores. Above 10^4^ Hz, we noticed from the PSD a drop in the noise due to the 10 kHz low pass filter, which is part of our current amplifier circuitry.

### Verification of RPS events arise from single rNMP molecules

**Fig. 4:**
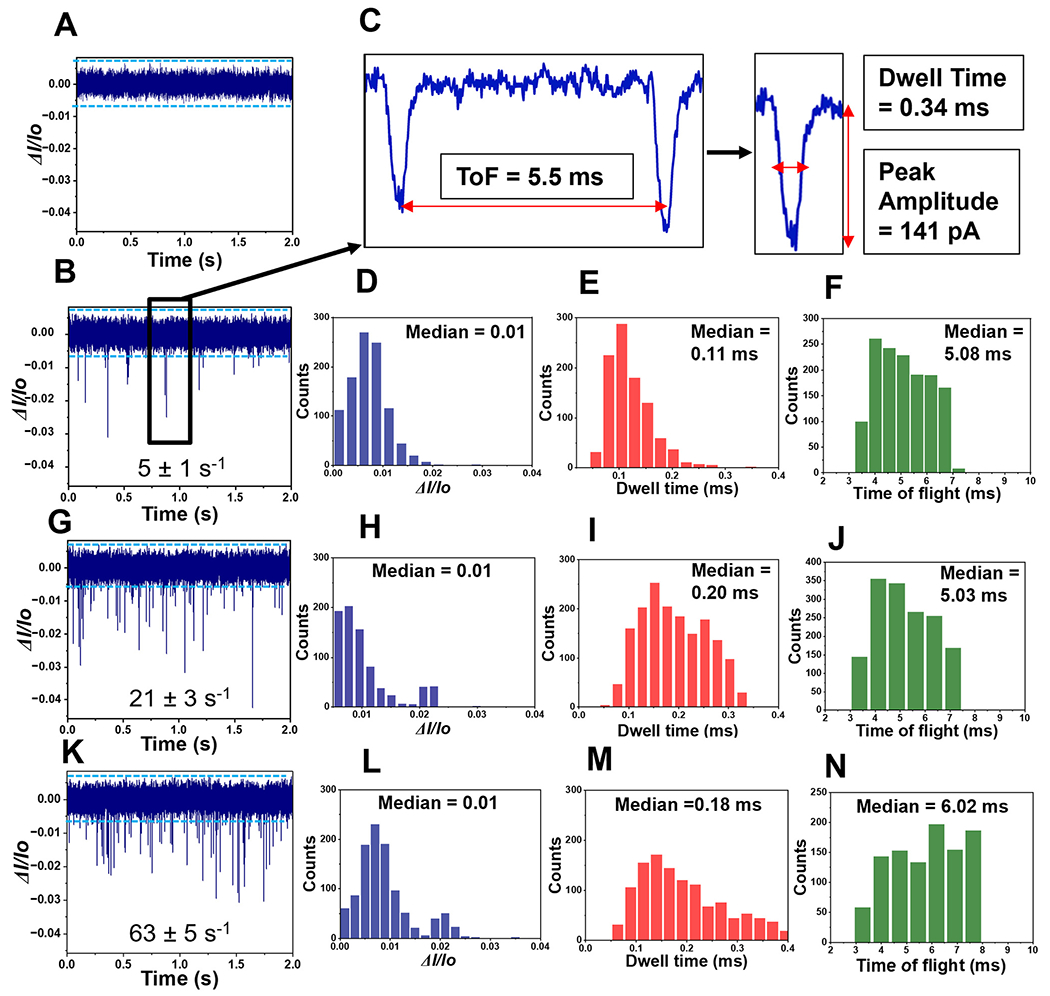
Label-free detection and identification of rNMPs using the dual in-plane nanopore sensor. RPS trace data for three different concentrations rCMP samples with each concentration run in different device (A). The first current trace is for the blank, which contained only 1× NEBuffer 3 (pH = 7.9). The blue line represents amplitude threshold condition. (B) RPS trace of 1 nM rCMP using the dual in-plane nanopore sensor with a carrier electrolyte of 1× NEBuffer 3 (2 s; total run was 2 min). (C) Expanded view of a single paired peak (see panel (B)) for rCMP showing the ToF, peak amplitude (absolute value), and dwell time for a single event of the pair. (D–F) Histograms for the peak Δ*I*/*I*_0_, dwell time, and the ToF for 1 nM rCMP RPS data. (G) RPS current trace data for 2 s of a 2 min run using 10 nM rCMP in 1× NEBuffer 3. (H-J) Histograms for the peak Δ*I*/*I*_0_, dwell time, and ToF for 10 nM rCMP RPS data. (K) RPS current trace for a 2 min run (shows expanded view of 2 s) for 100 nM rCMP in 1× NEBuffer 3. (L–N) Histograms for the peak Δ*I*/*I*_0_, dwell time, and ToF for the 100 nM rCMP RPS data. In all cases, a COP/COC dual in-plane nanopore sensor was used with a 10 μm nano-flight tube length using a driving voltage of 2.5 V at the sample inlet. All data was subjected to a 100 Hz high pass filter and a 10 kHz low pass filter. The electrophoresis was operated with the sample inlet being the anode and the receiving reservoir being the cathode. Thus, the electrokinetic motion of the rNMPs was in the same direction as the EOF. The numbers shown in panels B, G and K represent the capture rate (s^−1^) for each rCMP concentration.

To ensure that the observed RPS events generated by our in-plane nanopore were from single rNMP molecules, we performed experiments in which different concentrations of rCMPs were analyzed. We first ran a blank (1× NEBuffer 3) to set the threshold condition and ensure no false positive events were observed for our sensor (see Fig. 4A). Then, different rCMP concentrations were analyzed (1, 10, 100 nM; see Fig. 4B, G, and K), respectively. Clearly the capture rate increased with increased concentration of rCMP. We plotted rCMP concentration *versus* the single rCMP capture rate (s^−1^) and found this to yield a correlation coefficient of 0.97.

For this RPS data, we built histograms for the normalized peak amplitude (Δ*I*/*I*_0_; event amplitude normalized with respect to the open pore current of the device), event dwell time, and the ToF for each concentration (see Fig. 4D–F – 1 nM; Fig. 4H–J – 10 nM; and Fig. 4L–N – 100 nM). As seen from this data, minimal changes in Δ*I*/*I*_0_ and the event dwell time were observed over the concentration range of 1–100 nM, indicating that we are monitoring the same molecular entity. For example, changes in Δ*I*/*I*_0_ may be indicative of dimers or other higher order aggregates of rCMP forming at higher concentrations. Inspection of the ToF data showed that at 100 nM, the ToF was slightly higher compared to the 1 and 10 nm solutions, which may indicate faster migrating entities. However, at 100 nM, there is a higher probability of multiple occupancy within the flight tube that can induce biases into the ToF determination.^38^

We should note that we also have reported on a COMSOL simulation of our dual in-plane nanopore sensor and found that upon passage of a particle of 1 nm diameter the in-plane nanopore with an effective diameter of 10 nm produced a measurable peak amplitude of 2 nA.^38^ In addition, the amplitude increased with the decrease of the pore diameter.

### RPS detection and identification of a mixture of rNMPs

**Fig. 5:**
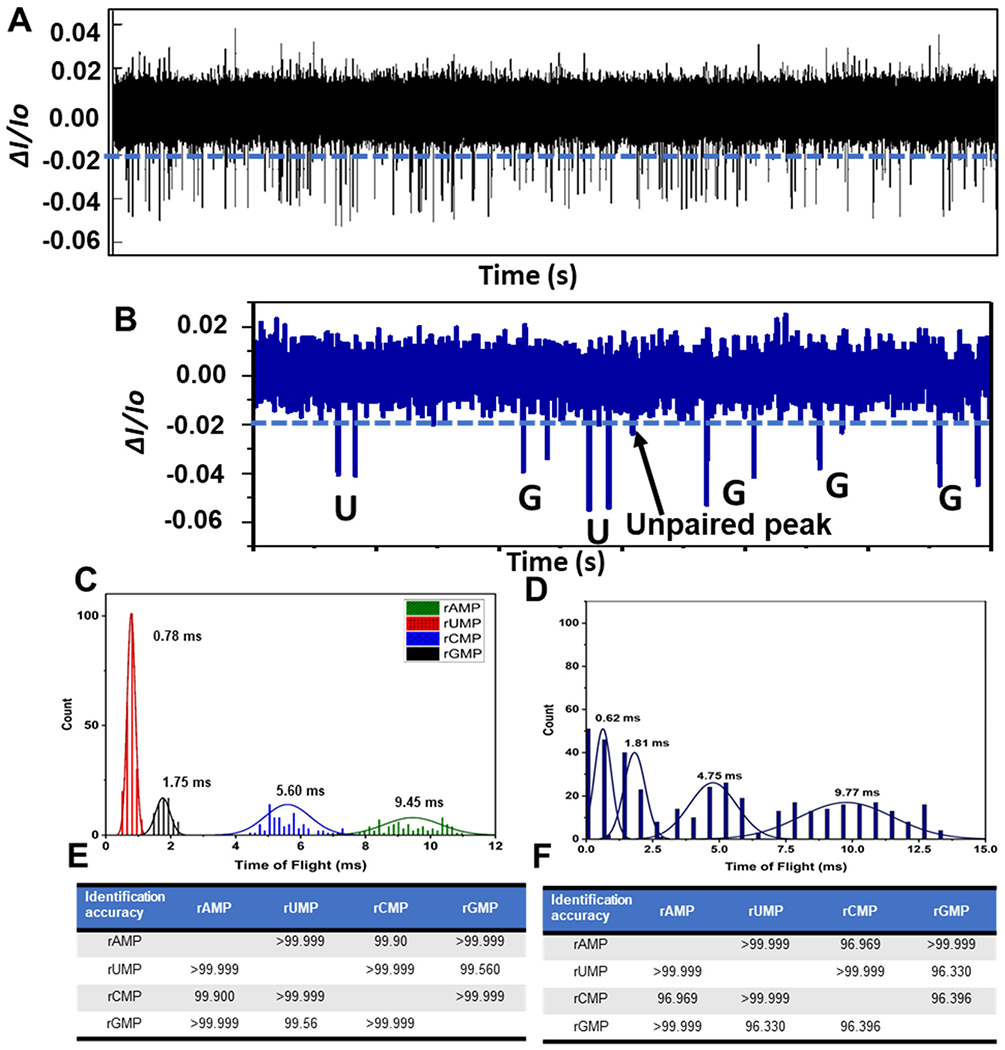
Label-free detection and identification of rNMPs using the dual in-plane nanopore sensor. (A) RPS trace data for an equimolar mixture of the rNMPs; total concentration = 10 nM. The blue line represents amplitude threshold condition. Even though some positive polarity events were visible, they were not scored as events because they did not exceed the time threshold condition for scoring as a RPS event, which was set at 0.1 ms (equal to the low pass filter bandwidth; 1/10 kHz). (B) RPS trace (120 ms) for a section of the data shown in (A) with the rNMPs called based on their ToF only in this case. There was a single non-paired event in this trace data. (C) Histograms of the ToFs for the rNMPs acquired using the dual in-plane nanopore sensor. In this case, each rNMP was run in a separate device. (D) Histograms of the ToFs for rNMPs acquired using the dual in-plane nanopore sensor. For this example, all rNMPs were run in a single device. (E) Identification accuracies of rNMPs calculated from Gaussian peak overlaps in the ToF data. For this data, each rNMP was run separately in a different device. (F) Identification accuracies for the rNMPs, which were run together in a single dual in-plane nanopore sensor device. In all cases, a COP/COC dual in-plane nanopore sensor was used with a 10 μm nanoflight tube length and 1× NEBuffer at pH 7.9 using a driving voltage of 2.5 V. In (E) and (F), the histograms were fit to Gaussian functions and each bin width represented 0.3 ms. All data was subjected to a 100 Hz high pass filter and a 10 kHz low pass filter.

We next performed RPS analysis for the detection and identification of a mixture of the four rNMPs with a total rNMP concentration of 10 nM. Here we used a COP/COC hybrid device, which was UV/O_3_ activated for 3.5 min. Fig. 5A shows the RPS trace data for a mixture of the four rNMPs and in Fig. 5B is shown a 120 ms section of the data taken from Fig. 5A, which shows 6 peak pairs with each peak pair in this section of data identified as either a rUMP or rGMP based on the ToF only. As can be seen in this RPS data, only one unpaired peak was found.

We next generated histograms for the ToFs from the data secured by running each rNMP separately using a different dual in-plane nanopore sensor for each ribonucleotide and a mixture of the rNMPs taken from Fig. 5A (see Fig. 5C and D, respectively). Comparison of Fig. 5C and D indicate that the separation between the ToF histograms seemed better for the rNMPs run separately compared to the mixture analysis. Careful inspection of Fig. 5C and D also indicated that the average ToF for each rNMP were similar whether the rNMPs were analyzed individually or as a mixture. The widths of the histograms were slightly wider for the mixture due to relaxation of criteria (III) for identifying peak pairs (*i.e.*, the maximum ToF is 1.5 × ToF for the corrected fluorescently labeled rNMP).

The ToF order at an electric field strength in the flight tube of 2000 V cm^−1^ (80% of applied voltage dropped across 10 μm flight tube) was rUMP < rGMP < rCMP < rAMP (see Fig. 5C or D). The observed ToF order was similar to the order we observed for nanoscale electrophoresis of ATTO-532 labeled rNMPs.^44^ Moreover, the peak variances of dye-labeled rAMP and rCMP were larger than those for rUMP and rGMP with similar observations observed here. This indicated that possible wall interactions were more prevalent for rAMP and rCMP compared to rUMP and rGMP. Furthermore, rAMP and rCMP showed longer ToF values compared to rUMP and rGMP, which again pointed to more wall interactions for rAMP and rCMP. These wall interactions can give rise to chromatographic effects in addition to electrophoretic effects accounting for differences in the apparent mobility for the four rNMPs.

The ToF histograms of the rNMPs were fit to Gaussian functions and the identification accuracies were determined from these Gaussian functions and are shown in Fig. 5E and F for the individual rNMPs and the mixture, respectively. The identification accuracy is defined as the amount of overlap between two adjacent Gaussian fits to the histograms of the rNMPs’ ToFs.^72^ The average identification accuracy for the experiment in which the rNMPs were run individually using separate devices was found to be 99.9% while for the mixture analysis, the identification accuracy was 98.3%.

Our previous work has shown that the device-to-device variation of the open pore current, which is determined primarily by the variation of the nanopore size, were 5% and 15% for the nanosensors fabricated by injection molding and NIL, respectively.^54^ Such low variation in the nanopore size further indicates that the device-to-device variation of the nanochannel would be negligible because the nanochannel is one order of magnitude larger than the nanopore.

### Additional data secured from current transient events and PCA

**Fig. 6:**
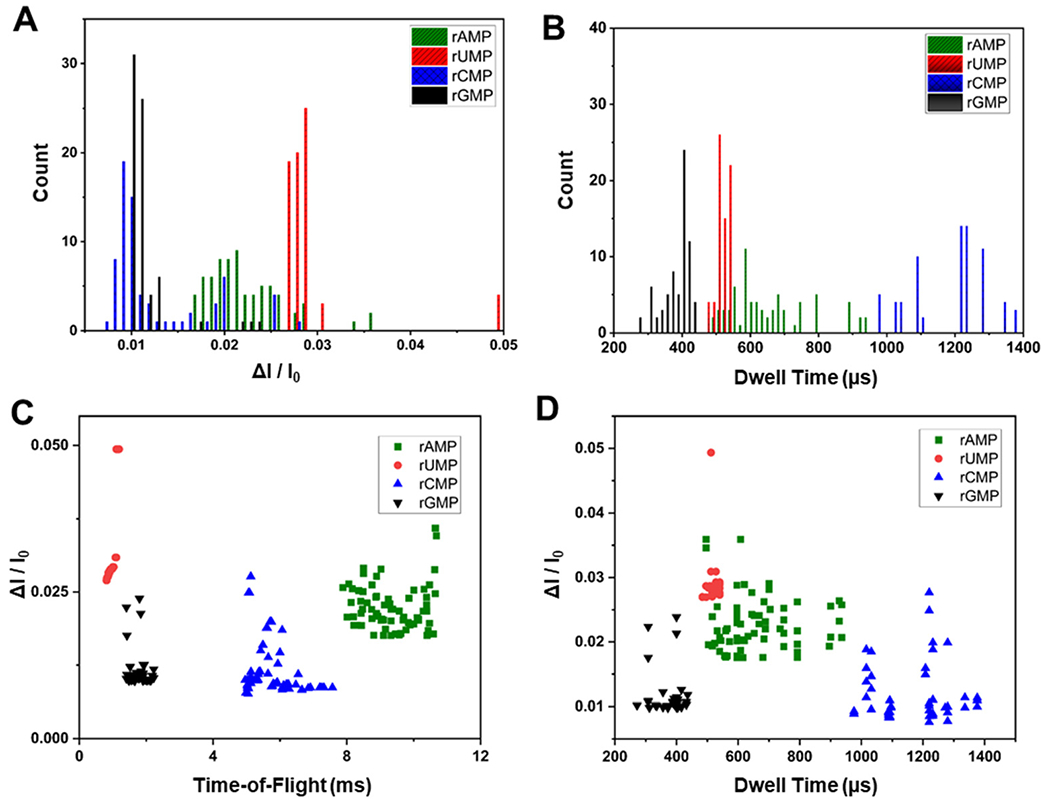
Identification of rNMPs using dual in-plane nanopore sensor with a 10 μm long nano-flight tube and COP/COC device. Histograms of (A) normalized peak amplitude and (B) dwell time for the rNMPs. Scatter plots of nucleotide translocation events showing (C) normalized peak amplitudes *versus* ToF, and (D) normalized peak amplitude *versus* dwell times for the rNMPs. Data were acquired with 1× NEBuffer 3 (pH.7.9) using a bias voltage of 2.5 V and a 10 μm long flight tube. The rNMP concentration was 10 nM.

The data secured from our dual in-plane nanopore sensor is not just the ToF, but also the dwell time, and the normalized current transient amplitude from each of the two pores in the series. Fig. 6A and B show histograms for Δ*I*/*I*_0_ and dwell times from transient current events acquired using the dual in-plane nanopore sensor with a 10 μm long flight tube for rAMP, rUMP, rCMP and rGMP. As seen from both histograms in Fig. 6A and B, significant overlap was apparent in the data for each rNMP.

We also generated scatter plots for Δ*I*/*I*_0_ *versus* ToF and Δ*I*/*I*_0_ *versus* dwell time, which are shown in Fig. 6C and D, respectively. As seen from Fig. 6C, there was slight overlap in the ToF for rGMP and rUMP, but inclusion of the Δ*I*/*I*_0_ values in the scatter plot provided better discrimination between these two rNMPs. In the case of Δ*I*/*I*_0_ *versus* dwell time there was no clear discrimination between the rNMPs.

We next performed PCA on the RPS data shown in Fig. 5 and included 5 variables (Δ*I*/*I*_0_ for pore 1, Δ*I*/*I*_0_ for pore 2, dwell time for pore 1, dwell time for pore 2, and the ToF) in the analysis. PCA transformed the original variables into a new set, where: (i) the new variables (principal components) were uncorrelated; (ii) the new variables accounted for all meaningful information (variance) in the original variable set; (iii) the number of new variables matched the number of original variables; (iv) the maximum amount of variance was assigned to the first new variable, remaining variance to second new variable, *etc.*; and (v) the new variables were linear combinations of the original variables. We also included a *K*-means clustering analysis into the PCA, an iterative method of partitioning all current transient events (284 in this case for the training set consisting of 71 RPS events for each rNMP) into 4 clusters.

**Fig. 7:**
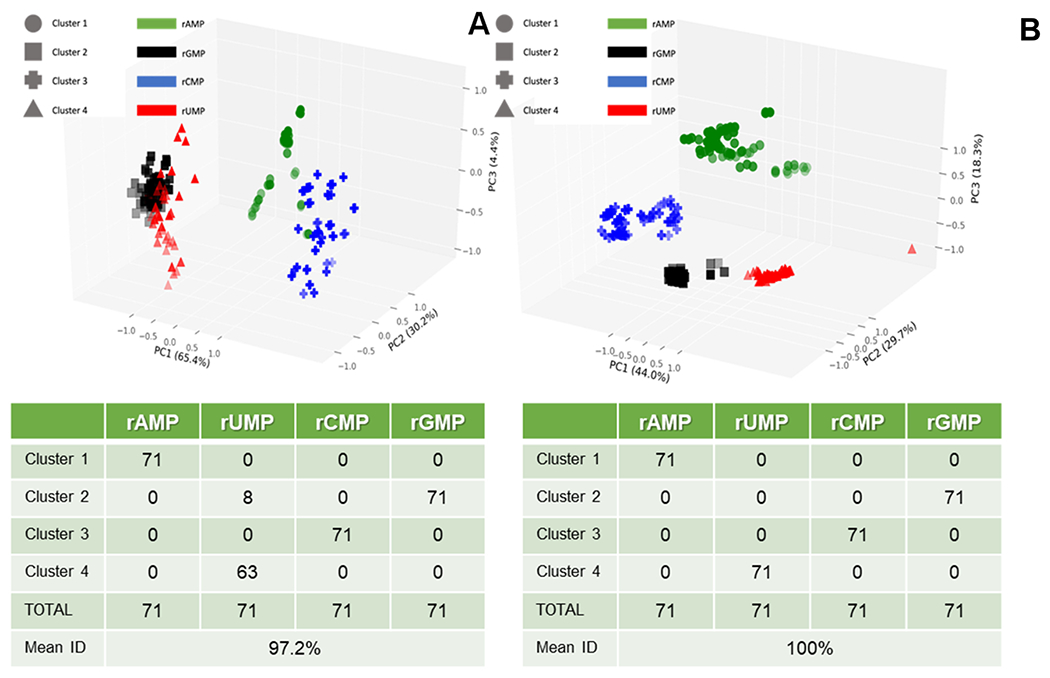
Principal component analysis (PCA) with *K*-clustering of the dual in-plane nanopore sensor data shown in Fig. 5. (A) Three-dimensional plot of *K*-mean clusters, which was generated using 3 variables (pore 1 dwell time, pore 2 dwell time, and ToF with 3 PCA variables (PC1 = 65.4%, PC2 = 30.2%, and PC3 = 4.4% of variance)). The accompanying table shows the 4 clusters and the classification of the 4 rNMPs into each of the four clusters. (B) Three-dimensional plot of *K*-mean clusters, which was generated using 5 variables including pore 1 dwell time, pore 2 dwell time, ToF, pore 1 normalized amplitude, pore 2 normalized amplitude with 3 PCA variables (PC1 = 44.0%, PC2 = 29.7%, PC3 = 18.3% variance). The table shows the classification accuracy, which in this case was 100%.

Fig. 7A shows a three-dimensional plot of the *K*-means clusters generated using only the time variables of the dual in-plane nanopore RPS data including the dwell times for pores 1 and 2, and the ToF data. As seen, three PCA variables were produced with their contribution to the variance ranging from 4.4% to 65.4% (total variance = 100% in these three PCA variables). However, the average identification accuracy for all four rNMPs was 97.2% when using the validation data set. When the same analysis was performed with the amplitude features added (Δ*I*/*I*_0_ one (pore one) and Δ*I*/*I*_0_ two (pore two)) to the time features, the average identification accuracy was 100%. The first three PCA components are plotted in Fig. 7B, which shows that the four rNMPs are all grouped correctly. Using Δ*I*/*I*_0_ along with time features in the PCA provided sufficient separation between the rNMPs to allow automated partitioning with 100% accuracy in each group assignment compared to using only the time related RPS variables. Finally, we should note that the dwell time and ToF values may be related to each other, but scaling factors make them somewhat orthogonal (*i.e.*, channel *versus* pore size). For example, the small size of the in-plane pore (effective diameter ~8 nm following thermal fusion bonding) compared to the flight tubes (50 × 50 nm) provides a larger electric field strength within the in-plane pores, more electrical double layer overlap giving rise to slight parabolic flow as opposed to plug-like flow in the nanochannel flight tube, and higher opportunity for wall adsorption/desorption effects.^38,45^

## Conclusion

In this work, we demonstrated the detection and identification of rNMPs using thermoplastic (PMMA or COP) dual in-plane nanopore sensors. An important attribute of these in-plane nanopore sensors is that a relatively large pore size can be used to detect even small molecules, like the rNMPs, compared to the out-of-plane pores due to the mechanical stability of the in-plane pores and the dielectric properties of the plastic. This advantage allows for relaxing stringent ion beam milling conditions during pore fabrication. The sensor contained a nano-electrophoresis flight tube flanked on either end with in-plane nanopores that could provide start/stop signals *via* RPS to deduce the apparent mobility from the ToF data using non-labeled targets. A nano-flight tube length of 10 μm was used to provide high identification efficiency of the rNMPs because of a combination of electrophoresis and chromatographic effects.^44,45^ Moreover, the capture rate of the dual in-plane nanopore sensor was found to increase when using a tapered interface between the micro- and nanofluidic networks compared to a blunt end geometry, which was verified with COMSOL simulations and agreed to previous literature findings.^56,73^ With the tapered geometry, we could detect RPS signals when the rNMP mass load into the sensor was 3.9 fg.

Unique to this sensor format is the number of variables that can be used for single molecule identification, namely the ToF, normalized current transient amplitude for pores 1 and 2, and the dwell times measured from pores 1 and 2. When using all of the RPS variables and PCA, 100% identification accuracy was generated for the rNMPs compared to 94% for the dNMPs when using only the ToF data.^38^ Finally, because we are using thermoplastics for the dual in-plane sensor, the devices can be mass produced for commercial applications using nano-injection molding.^54^

An ultimate goal of this technology is to perform RNA sequencing at the single-molecule level using an exonuclease, such as XRN1, to generate rNMPs from single intact RNA molecules. In this case, the spacing between single rNMPs within the dual in-plane nanosensor, which includes the two in-plane pores flanking the nanometer flight tube, is determined by the clipping rate of the exonuclease enzyme and not the concentration of the rNMPs as is the case for the sensor shown in Fig. 1 that is based on stochastic sensing of single molecules from the microchannel into nanochannels.

## Materials and methods

### Materials and reagents

Please see the ESI† material for a description of all chemicals and reagents used in these studies.

### COMSOL simulations

COMSOL simulations were performed using COMSOL Multiphysics 5.5 software to evaluate electric field strengths throughout the fluidic circuit associated with the dual in-plane nanopore sensor.

### Fabrication and assembly of nanofluidic devices *via* NIL

Nanofluidic devices were fabricated in thermoplastics using a method previously reported by our group.^39^ Please see the ESI† and Fig. S1 for more information.

### Atomic force microscopy

To obtain metrology data on the nanopores, AFM (SPM HT-9700, Shimadzu Corporation, Kyoto, Japan) analysis was carried out. The probe used for imaging was a Super Sharp Silicon tip (Nanosensors, Switzerland) with a tip radius <2 nm, half cone angle of 10°, aspect ratio 4 : 1 at 200 nm from the tip apex, and a frequency of 300 kHz. A dynamic scanning mode was used for imaging with a scanning frequency of 0.5 Hz. The acquired images were analyzed using SPM Manager v4.76.1 software.

### Scanning electron microscopy

SEMs of the dual in-plane nanopore sensors were acquired using a FEI VERSA 3D Dual-beam field emission/low vacuum SEM. A 2 nm thin conductive iridium layer was sputter coated onto the devices using an EMS 150ES sputter coater before SEM imaging. All images were acquired using 5.0 kV accelerating voltage and 8.7 mm working distance. The SEM images of the Si master molds were collected using a Quanta 3D Dual-beam FEI FIB-SEM and were analyzed using the instrument’s software and Image J.

### Translocation of rNMPs through the dual in-plane nanopore sensor

Translocation experiments were performed for rNMPs in PMMA/COC or COP/COC dual in-plane nanopore sensors bonded at 170 psi for 15 min. Briefly, after methanol/water priming, 1× NEBuffer 3 (100 mM NaCl; 50 mM Tris-HCl; 10 mM MgCl_2_; 1 mM DTT; pH 7.9 at 25 °C) was introduced into the device. Finally, 10 or 100 nM of rNMPs seeded into 1× NEBuffer 3 was introduced into one of the reservoirs of the sensor. The sensors were placed in a Faraday cage and Ag/AgCl electrodes were immersed into the reservoirs of the sensor. A potential of 2.5 V was applied by serially connecting a 1.5 V battery to the Axopatch Digidata 1440B circuit and data was acquired at a sampling frequency of 250 kHz, a head stage configuration of *β* = 0.1, gain = 1, and a low pass filter of 10 kHz. Data were collected for a period of 10 min and Clampfit 11.1 software was used for data acquisition and analysis.

### Principal component analysis (PCA)

Principal component analysis (PCA) was performed in R version 4.1.3, and a score plot was generated using the “factoextra” package. Additional PCA and *K*-means clustering analyses were performed in Python 3 with the “Scikit-learn” package. The data were centered and scaled before PCA transformation.

### UV/O_3_ and O_2_ plasma activation of thermoplastics

Nanofluidic devices imprinted into PMMA of COP were exposed to UV light (20 mW cm^−2^) for 3, 5, 10 and 15 min. Microscopic images were captured using a Keyence microscope after each exposure. In some cases, O_2_ plasma activation of the polymer surface was also undertaken at 50 W for 1 min.

## Supplementary Material

SI

## Footnotes

† Electronic supplementary information (ESI) available. See DOI: https://doi.org/10.1039/d3lc01062g
