# Supplementary material for: Detection and identification of single ribonucleotide monophosphates using a dual in-plane nanopore sensor made in a thermoplastic via replication
Source: Lab Chip. Author manuscript; Available in PMC 2024 Jul 17. (PMC11091956; doi:10.1039/d3lc01062g)
Supplement: SI [file NIHMS1989805-supplement-SI.pdf]

Electronic Supplementary Material (ESI) for Lab on a Chip.
This journal is © The Royal Society of Chemistry 2024

Supporting Information

Detection and Identification of Single Ribonucleotide Monophosphates using a Dual In-Plane Nanopore Sensor Made in a Thermoplastic via Replication

Chathurika Rathnayaka,[1,2‡] Indu A. Chandrosoma,[1,2‡] Junseo Choi,[2,3‡] Katie Childers,[1,2‡] Maximillian Chibuike,[1,2] Khurshed Akabirov,[1,2] Farhad Shiri, [1,2] Adam R. Hall,[2,4*] Collin McKinney,[6] Matthew Verber,[6] Sunggook Park,[2,3*] and Steven A. Soper[1,2,7,8,9*]

[1]Department of Chemistry, The University of Kansas, Lawrence, KS 66045
[2]Center of Biomodular Multiscale Systems for Precision Medicine
[3]Department of Mechanical Engineering, Louisiana State University, Baton Rouge, LA 70803
[4] Virginia Tech-Wake Forest School of Biomedical Engineering and Sciences, Wake Forest School of Medicine, Winston Salem, NC 27101
[6]Department of Chemistry, University of North Carolina, Chapel Hill, Chapel Hill, NC 27599
[7]Department of Mechanical Engineering, The University of Kansas, Lawrence, KS 66045
[8]Bioengineering Program, The University of Kansas, Lawrence, KS 66045
[9]KU Cancer Center, University of Kansas Medical Center, Kansas City, KS 66160

*Authors to whom correspondence should be addressed.

‡ These authors contributed equally to this work.

Nanoimprint lithography (NIL) fabrication of dual in-plane nanopore sensors. Fabrication of dual in-plane nanopore sensors is shown in **Figure S1A.** Si wafers that were 100 nm thick and possessed a silicon nitride (Si_3N_4) layer on each side were used for fabricating the Si master mold. Microchannels were fabricated using photolithography and wet-chemical etching. This was done by placing a 1.3 µm thick S1813 photoresist layer spin-coated at 4,000 rpm for 60 s on the Si wafer followed by baking at 115°C for 60 s. Photolithography was performed using the appropriate photomask and a UV exposure station (Quintel) in a class 100 cleanroom. UV exposure was carried out at 130-140 mJ/cm^2 with a post-exposure bake at 95°C for 60 s. The wafer was then subjected to development using a MF319 developer followed by washing with deionized water. The exposed Si_3N_4 layer was etched using ICP-DRIE (Plasmalab System 100, Oxford Instruments, Abingdon, UK). The wafer was then placed in a 40 wt% KOH bath with IPA (5 % v/v; 70°C). After 25 min etching to form 5 µm deep microchannels, the wafer was removed from the KOH bath, rinsed with water, and dried with N_2. Prior to FIB milling, the Si_3N_4 layer was removed using a dilute HF solution. The nanostructures comprising the sensor were fabricated using FIB (Quanta 3D Dual Beam system, FEI, Hillsboro, OR), which was performed at a beam voltage and current of 30 kV and 10 pA, respectively, in a bitmap mode.

The Si master mold with the appropriate microstructures and nanostructures was used to produce a resin stamp using a UV resin solution (70 wt% TPGDA, 28 wt% TMPTA, and 2 wt% photoinitiator). Drops of the UV-resin were dispensed onto the Si master mold and a flexible PET sheet coated with an adhesive layer (NOA72) was then slightly pressed against the liquid drop and used as a backbone for the resin stamp. Residual resin solution and air bubbles were gently removed. During the curing process, the sample was exposed to a flash-type UV light (250-400 nm) for 20 s at an intensity of ~1.8 W/cm^2 using UV nanoimprint lithography, NIL (Eitre6, Obducat, Lund, Sweden). After UV-curing, the molded UV-resin/PET backbone was demolded from the Si master. See **Figure S1B** for a process flow diagram.

Nanopore devices were thermally imprinted into the appropriate plastic substrate using thermal NIL (Nanonex 2500, Monmouth Junction, NJ).[1] The optimized imprinting conditions were 145°C, 300 psi, and 5 min for PMMA nanofluidic devices, and 130°C, 300 psi, and 5 min for COP devices. Imprinted nanofluidic devices were then characterized using scanning electron microscopy (SEM) and atomic force microscopy (AFM).

NIL produced devices were sealed using a COC 8007 cover plate (see **Figure S1D**) via thermal fusion bonding. Both the substrate and cover plate were exposed to UV light (20 mW/cm^2) for 3 min or an O_2 plasma (1 min) to activate the surface before thermal fusion bonding. The device was assembled using the Nanonex 2500 nanoimprint lithography (NIL) machine.

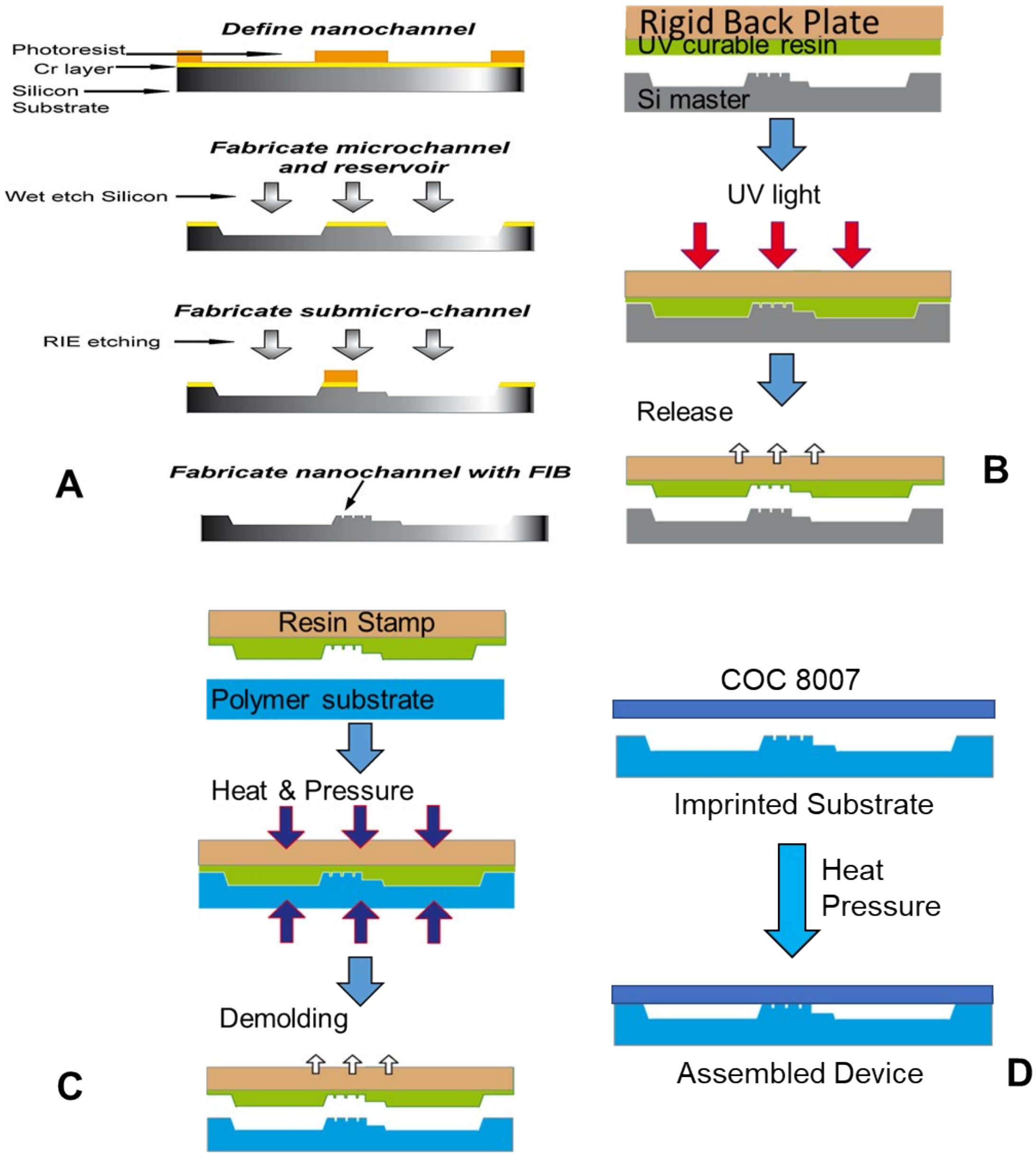

Figure S1. Fabrication steps of nanofluidic devices using NIL. (**A**) Schematic showing the production of the Si master mold, which used a combination of photolithography (microstructures) and focused ion beam milling for the nanostructures. (**B**) Process strategy for making resin stamps from the Si master mold using UV-NIL. (**C**) Thermal NIL for producing finished devices in the appropriate thermoplastic using the resin stamp. (**D**) Strategy for thermal fusion bonding a low Tg cover plate (COC 8007) to the imprinted device, which possessed a higher Tg than the cover plate.

Table S1. Physical dimensions of the dual in-plane nanopore sensor and the variables used for the COMSOL simulation of this device.

Designed Channel Dimensions	
	Width × Depth × Length
Microchannels	50 μm × 5 μm × 7.5 mm
Tapered Funnels	5 μm × 1 μm × 9 μm
Nanopores	10 nm × 10 nm × 12 nm
ToF Flight Tube	50 nm × 50 nm × 5 μm
**COMSOL Simulation Variables**	
Buffer Solution	1 M KCl
Conductivity	10.9 S/m
Equation	Ohm's Law
Applied Voltage	-2.5 V
Diffusion Constants	2.03×10^{-9} m^2/s (Cl-)<br>1.95×10^{-9} m^2/s (K+)
Charge Number	-1 (Cl-)<br>+1 (K+)

Chemicals and Reagents. Chemicals and other materials were obtained from the following sources and used as received. Silicon (Si) 〈100〉 wafers were purchased from University Wafers (Boston, MA). SU-8 2005 photoresist was obtained from MicroChemicals (Germany). SU-8 developer was obtained from Advanced Materials (Westborough, MA). Potassium hydroxide (KOH) pellets were received from Fisher Scientific (Waltham, MA). Isopropyl alcohol (IPA) and hydrofluoric acid (HF) were purchased from Sigma-Aldrich (St. Louis, MO). PMMA was received from ePlastics (San Diego, CA). Nano-Strip solution was purchased from Electronic Chemicals (Houston, TX). Polyethylene terephthalate (PET) sheets with a thickness of 250 μm were secured from Goodfellow (Coraopolis, PA). Cyclic olefin copolymer (COC 8007) was purchased from TOPAS Advanced Polymers (Florence, KY). TPGDA (Tri(propylene glycol) diacrylate, Trimethylolpropane triacrylate (TMPTA), 2,2-Dimethoxy-2-phenylacetophenone (photo initiator), and 2-Hydroxy-2-methylpropiophenone (photo initiator) were purchased from Sigma-Aldrich (St. Louis, MO). Polyurethane resin (PUA) was obtained from Chansang Co. Perfluoropolyether (MD 700) was obtained from Solvay (Alorton, IL) and NOA72 was obtained from Norland Products, Neuchâtel, Switzerland. Adenosine 5'-monophosphate disodium salt, guanosine 5'-monophosphate disodium salt, cytidine 5'-monophosphate disodium salt, and uridine 5'-monophosphate disodium salt were obtained from Sigma-Aldrich (St. Louis, MO). Molecular biology grade water was secured from Thermo Fisher (Waltham, MA). Cyclic olefin polymer sheets were obtained from Zeonex (San Jose, CA).

A

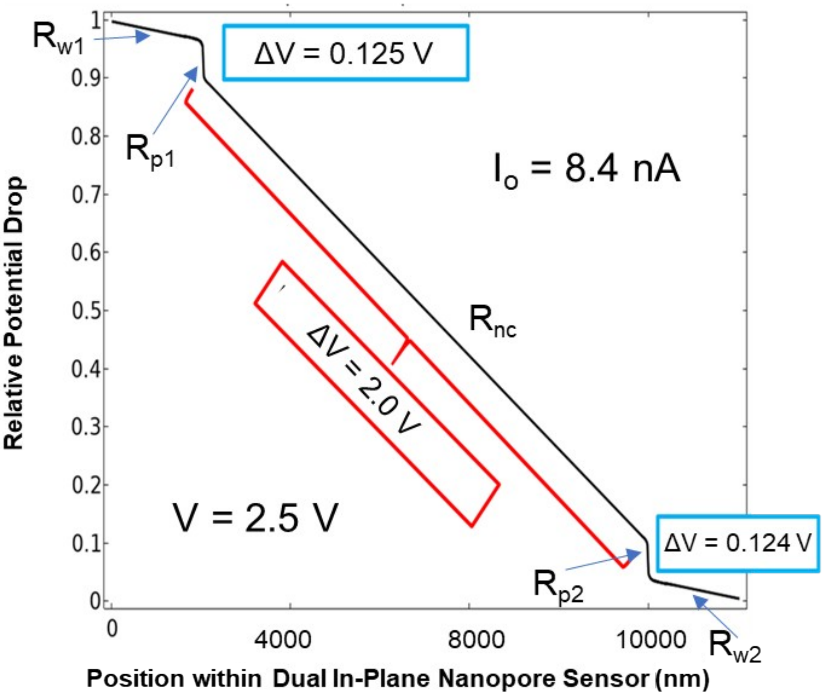

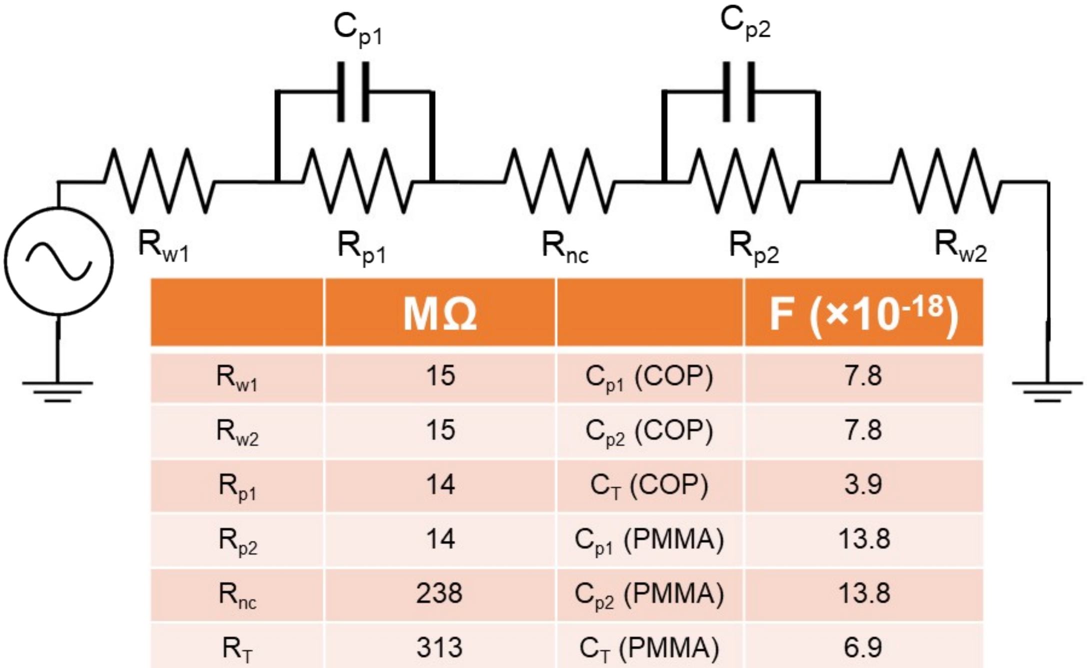

B

	MΩ		F (×10^{-18})
R_{w1}	15	C_{p1} (COP)	7.8
R_{w2}	15	C_{p2} (COP)	7.8
R_{p1}	14	C_T (COP)	3.9
R_{p2}	14	C_{p1} (PMMA)	13.8
R_{nc}	238	C_{p2} (PMMA)	13.8
R_T	313	C_T (PMMA)	6.9

C

Figure S2. (**A**) Fluidic circuit showing the relative voltage drop across each element of the circuit (not included is the microfluidic network). The numeric value is deduced from the percentage of voltage drop (V) across each element of the circuit and the applied voltage across the fluidic circuit. The open pore

current (I_0) was determined using a carrier electrolyte consisting of 1× NEBuffer 3 (pH = 7.9). (**B**) Equivalent electrical circuit for the fluidic circuit shown in (**A**), which consists of a series of resistors and two capacitors in parallel. The fluidic circuit has a voltage applied across it, which in this case was 2.5 V. (**C**) Table showing the values of the various resistances and capacitances of the components making up the fluidic circuit.

In **Figure S2A** is shown the relative voltage drop through the dual in-plane nanopore sensor along with the open pore current at an applied voltage of 2.5 V while in **Figure S2B** is shown the equivalent electrical circuit of the nanofluidic device that consisted of several resistors in series with a capacitor in parallel to each pore resistance. To determine the fluidic circuit's bandwidth, f_C, we needed to calculate R_T and C_T (see equation 3 in the main text). R_T was determined from the 3 resistors in series, namely R_{w1}, R_{nc}, and R_{w2}, but excluded R_{p1} and R_{p2}. The value for each resistive element was deduced from the applied voltage (2.5 V) and the current through the system (8.4 nA) using Ohm's law. The values for the relevant elements are shown in Figure S2C with RT shown as well. For CT, we calculated the capacitance for each pore (CS or Cp1 and Cp2) using CT = $1/C_{p1} + 1/C_{p2}$; if $C_{p1} = C_{p2}$, then $C_T = C_p/2$. C_p for pore 1 or pore 2 was calculated using;

$$C_p = \frac{(\varepsilon_0 \varepsilon_r A_p)}{d} \quad \text{(S1)}$$

where d is the average spacing between the capacitor plates (10 nm), A_p is the area of the capacitor plates (4.0×10^{-15} m^2), ε_0 is the permittivity of free space (8.85×10^{-12} F/m), and ε_r is the material's relative dielectric (COP, ε_r = 2.2; PMMA, ε_r = 3.9). **Figure S2C** shows C_T values for PMMA and COP devices.

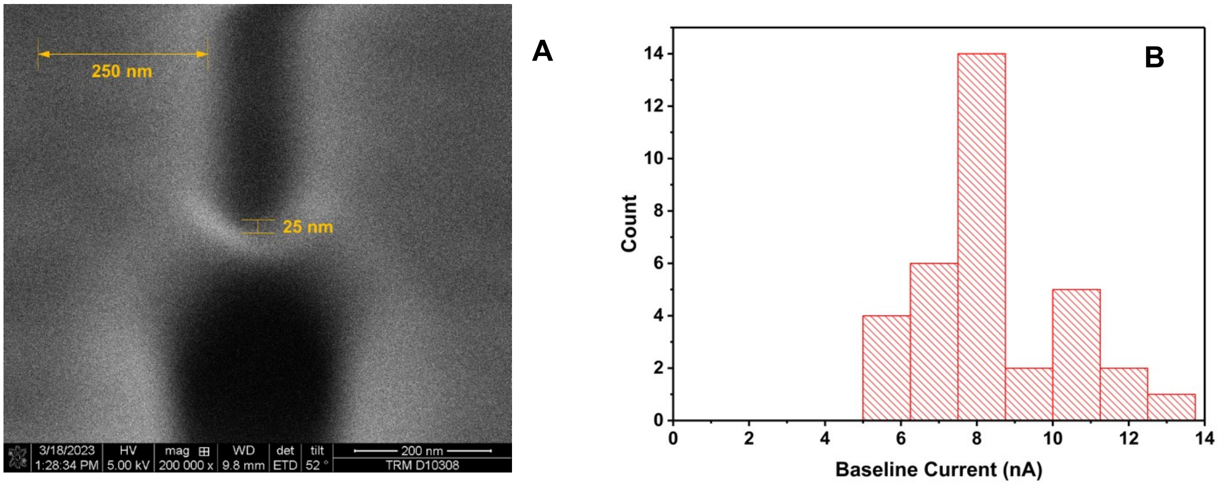

Figure S3. (**A**) High resolution SEM of an in-plane pore taken from the device shown in **Figure 1** of the main text. (**B**) Histogram of baseline current (*i.e.*, open pore current, I_0) of dual in-plane nanopore sensors used for rNMP translocation experiments (1× NEBuffer 3 at pH 7.9) and an applied voltage of 2.5 V (n = 34).

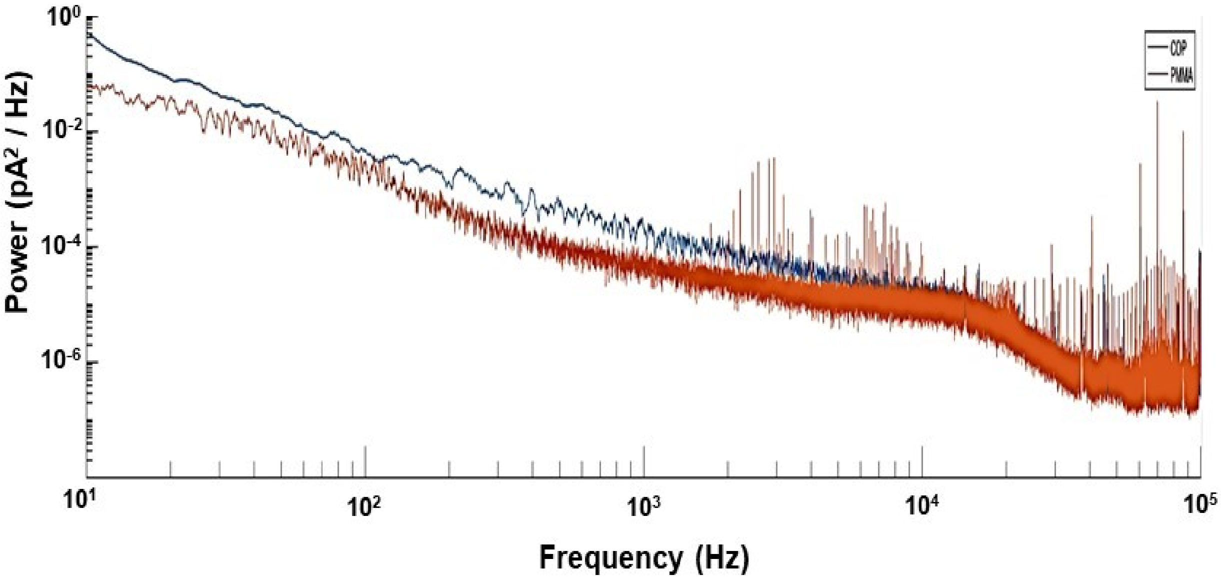

Figure S4. Power spectral density (PSD) for a COP and PMMA dual in-plane nanopore sensor device. In both cases, the PSDs were performed using 1× NEBuffer 3 (pH = 7.9) with an applied voltage to the device equal to 2.5 V. The data was subjected for both plastics to a 15-point moving average filter. The apparent peak in the PSD at $f > 10^4$ is due to interaction of the in-plane pore capacitance with that of the Axopatch current amplifier. Finally, the spiking present in the PSD for the PMMA device is likely due to signal aliasing – we are correcting for this in the future using the appropriate anti-aliasing filter.

References

1. Amarasekara, C. A.; Rathnayaka, C.; Athapattu, U. S.; Zhang, L.; Choi, J.; Park, S.; Nagel, A. C.; Soper, S. A., Electrokinetic identification of ribonucleotide monophosphates (rNMPs) using thermoplastic nanochannels. *Journal of Chromatography A* **2021,** *1638*, 461892.
